# Targeted silencing of the Aquaporin 2 gene of *Rhipicephalus (Boophilus) microplus* reduces tick fitness

**DOI:** 10.1186/s13071-015-1226-2

**Published:** 2015-12-02

**Authors:** Hala E. Hussein, Glen A. Scoles, Massaro W. Ueti, Carlos E. Suarez, Fatma K. Adham, Felix D. Guerrero, Reginaldo G. Bastos

**Affiliations:** Department of Veterinary Microbiology and Pathology, Washington State University, Pullman, WA 99164 USA; Department of Entomology, Faculty of Science, Cairo University, Giza, 12613 Egypt; Animal Disease Research Unit, USDA-ARS, Washington State University, 3003 ADBF, P.O. Box 646630, Pullman, WA 99164 USA; USDA-ARS, Knipling Bushland US Livestock Insect Research laboratory, 2700 Fredericksburg Road, Kerrville, TX 78028 USA; USDA-ARS Veterinary Pest Genomics Center, Kerrville, TX USA; School of Molecular Biosciences, Washington State University, Pullman, WA 99164 USA

**Keywords:** *Rhipicephalus* (*Boophilus*) *microplus*, Aquaporin, Tick fitness, RNA interference, Gene silencing, *Babesia bovis*

## Abstract

**Background:**

Ticks are blood-feeding arthropods that can affect human and animal health both directly by blood-feeding and indirectly by transmitting pathogens. The cattle tick *Rhipicephalus* (*Boophilus*) *microplus* is one of the most economically important ectoparasites of bovines worldwide and it is responsible for the transmission of the protozoan *Babesia bovis*, the etiological agent of bovine babesiosis. Aquaporins (AQPs) are water channel proteins implicated in physiological mechanisms of osmoregulation. Members of the AQP family are critical for blood-feeding arthropods considering the extreme osmoregulatory changes that occur during their feeding. We investigated the pattern of expression of a newly identified *AQP2* gene of *R. microplus* (*RmAQP2*) in different tick tissues and stages. We also examined *in vivo* the biological implications of silencing expression of *RmAQP2* silencing during tick feeding on either uninfected or *B. bovis*-infected cattle.

**Methods:**

*In silico* gene analyses were performed by multiple alignments of amino acid sequences and topology prediction. Levels of *RmAQP2* transcripts in different tick tissues and stages were analyzed by reverse transcriptase quantitative PCR. Patterns of expression of RmAQP2 protein were investigated by immunoblots. Gene silencing was performed by RNA interference and *in vivo* functional analyses carried out by feeding ticks on either uninfected or *B. bovis*-infected cattle.

**Results:**

*RmAQP2* transcripts were found in unfed larvae, engorged nymphs, and salivary glands and guts of partially engorged females; however, of all tick tissues and stages examined, RmAQP2 protein was found only in salivary glands of partially engorged females. *RmAQP2* silencing significantly reduced tick fitness and completely abrogated protein expression. The effect of *RmAQP2* silencing on fitness was more pronounced in females fed on a *B. bovis*-infected calf than in ticks fed on an uninfected calf and none of their larval progeny survived.

**Conclusions:**

Collectively, considering the gene expression and tick fitness data, we conclude that *RmAQP2* is critical for tick blood feeding and may be a suitable candidate target for the development of novel strategies to control *R. microplus* and tick-borne parasites.

**Electronic supplementary material:**

The online version of this article (doi:10.1186/s13071-015-1226-2) contains supplementary material, which is available to authorized users.

## Background

Aquaporins (AQPs) are a family of transmembrane proteins that form pores to transport water and small solutes across cellular membranes [1]. Members of the AQP family have been identified throughout the plant and animal kingdoms [2]. The structures of AQPs are highly conserved among species, consisting of six transmembrane domains that are connected by two intracellular loops and three extracellular loops. Two asparagine-proline-alanine (NPA) motifs are considered AQP signature motifs and are located at the protein portion that interacts to form a pore [1]. A total of 13 AQP members have been identified so far and classed into two subsets: those that are permeated by water and those that are permeated by water plus other small molecules, such as glycerol and urea [1, 2].

Numerous members of the AQP family have been identified in arthropods in the last few years due to the availability of several arthropod genomes and genetic information, such as transcriptomes and cDNA libraries [3]. Studies have shown that AQPs play a pivotal role in arthropods, especially in blood-feeding species, such as mosquitoes and ticks. In fact, blood-feeding arthropods have become a model for AQP research due to the critical relevance that osmoregulation plays during feeding [4]. Tick females can take on up to 100 times their body weight in blood, and concentrate the blood meal by returning approximately 75 % of the ingested water and ions via their saliva into the host [5]. Therefore, the osmoregulatory system of ticks is central for their parasitic life cycle and has implications for efficient feeding and subsequent production of viable offspring.

The cattle tick *Rhipicephalus* (*Boophilus*) *microplus* is one of the most economically important ectoparasites of bovines, as it is the main vector of the apicomplexan protozoan *Babesia bovis*, the etiological agent of bovine babesiosis [6]. Adult females *R. microplus* acquire *B. bovis* merozoites by ingesting blood from an infected bovine and pass the protozoan transovarially to their larval progeny, which then can transmit *B. bovis* sporozoites to naïve cattle during subsequent feeding [6–8]. In most endemic areas control of bovine babesiosis transmission relies mainly on targeting populations of *R. microplus* with acaricide treatments and to a lesser extent with the use of live attenuated vaccines [6–10]. However, the efficacy of commercial anti-tick vaccines is inconsistent in different regions of the world and the recent development of tick populations resistant to acaricides represents a serious threat to the cattle industry [11, 12]. Additionally, the re-emergence of *R. microplus* in areas that had been considered to be free of this tick, such as the regions outside the permanent quarantine zone in south Texas, USA, is causing concerns about the re-establishment of active *B. bovis* transmission in areas currently free of bovine babesiosis. Exposure of naïve cattle in these areas to *B. bovis* would lead to significant mortality since no herd immunity is present in the population.

Intake of blood meals leading to full engorgement of adult females and subsequent production of viable larval offspring are critical steps in the tick life cycle. A better understanding of mechanisms involved in these processes may lead to the identification of novel targets to control ticks and tick-borne parasites. In the present study we investigated the pattern of expression of a newly identified *AQP2* gene of *R. microplus* (*RmAQP2*). Our findings showed that *RmAQP2* is transcribed in unfed larvae, engorged nymphs, and salivary glands and guts of partially engorged females. However, among the tick tissues and stages analyzed, RmAQP2 protein was only found in salivary glands of partially engorged females. *In vivo* analyses demonstrated that silencing of *RmAQP2* with RNA interference significantly reduced tick fitness and this effect was even more dramatic in females fed on a calf during acute *B. bovis* infection than in ticks fed on an uninfected calf.

## Methods

### Cattle, ticks and protozoan

Holstein calves 3–4 months of age, tested negative for *B. bovis* by PCR [13] and cELISA [14], were used in this study. The animals were maintained according to protocols approved by the University of Idaho Institutional Animal Care and Use Committee. Ticks from our laboratory colony, *Rhipicephalus microplus* La Minita strain [15] were tested by sequencing the cytochrome oxidase 1 (*Cox1*) gene to confirm that they were of the same strain as those used to obtain the sequences from the *R. microplus* gene index project [16, 17]. For tick strain identification, genomic DNA was prepared from *R. microplus* male ticks. PCR using the *Cox1* external and nested primers (Table 1) and prepared with FastStart *Taq* reagents (Roche Diagnostics, Indianapolis, IN) was carried out under the following conditions for both rounds: 95 °C for 5 min; 35 cycles of 95 °C for 1 min, 65 °C for 1 min, and 72 °C for 2 min; final extension at 72 °C for 5 min. The nested PCR product was cloned into a pCR™4 TOPO**®** plasmid for sequencing (Invitrogen, Carlsbad, CA). The *Cox1* sequence obtained from our tick colony had 100 % identity with the mitochondrial genome sequence of the *R. microplus* Deutsch strain from Texas, USA [GenBank: KP143546], which was used to obtain the *RmAQP2* sequence, and confirming that our laboratory colony represents a prototypical strain of *R. microplus* [18].

**Table 1:** Primer purposes and sequences, gene region and PCR product size for *Rhipicephalus microplus Cox1* and *RmAQP2*

Primer purpose	Forward primer	Reverse primer	Gene region	Product size (bp)
*Cox1* amplification external	attttaccgcgatgaatatactc	gcaaggcctaaaaaatg	1 to 1298	1298
*Cox1* amplification nested primers	ctcaactaatcataaagacattgg	gctaatataattcctgttaaacctcc	21 to 1072	1052
*RmAQP2* dsRNA synthesis	aattcagcagcaggagaagc	cggcgtacaccaggtaaact	17 to 414	397
*RmAQP2* dsRNA synthesis	cctctcctcgtcggcctca	cggctaaaacgcaaaaaggt	614 to 1009	396
*RmAQP2* Real-time PCR	gtaagtcaccgcacagta	tacacaatagcgaggtt	349 to 453	105

To obtain unfed adult ticks for each experiment, approximately 40,000 larvae from 2 g of eggs were placed under a cloth patch on uninfected calves. On day 13–14, engorged nymphs were manually removed and held in an incubator at 25 °C with 96 % RH to molt to adults. After 2–3 days of incubation, freshly molted unfed adult females and males were sorted out and used for evaluation of gene expression. The first silencing experiment was carried out by placing injected ticks on a calf infected with *Babesia bovis*. The second silencing experiment was done on an uninfected calf. For the experiment with the *Babesia bovis* infected animal, the calf was injected with a stabilate containing approximately 1.4 × 10^8^*B. bovis*-infected erythrocytes (T2Bo strain) [19]. The infected calf was monitored daily for the presence of *B. bovis* in peripheral blood and clinical signs of babesiosis. Parasitemia of *B. bovis* in peripheral blood was examined by qPCR to amplify the single copy *msa-1* gene as previously described [13]. The *B. bovis*-infected animal presented clinical indications of acute *B. bovis* infection including a drop in PCV, fever and detection of parasites in peripheral blood by qPCR (data not shown).

### *In silico* analysis

Nucleotide sequence encoding for *RmAQP2* cDNA was obtained from the *R. microplus* gene index project [16, 17] [GenBank ID: KP406519]. Multiple alignments of amino acid sequences were generated using the Multiple Alignment Module of LaserGene of DNASTAR software® (DNASTAR, Inc.). The Simple Modular Architecture Research Tool (SMART) [20] and the Transmembrane Hidden Markov Model package 2 (TMHMM2) [21] were used to predict domains and signal peptides in the RmAQP2 protein sequences. The tools Transmembrane Protein Representation in 2 Dimensions (TMRPres2) [22] and Swiss-model [23] were used to create 2D and 3D (respectively) visual representations of the RmAQP2 protein for topology analyses.

### Reverse transcriptase real-time PCR

A reverse transcriptase quantitative real-time PCR (RT-qPCR) was standardized to assess the level of expression of *RmAQP2* in different tick tissues and stages. In order to make solid significant observations regarding gene expression, qPCR was designed using the pertinent requirements of the minimum information for publication of qPCR experiments [24]. Unfed larvae, engorged nymphs, unfed male ticks, and salivary glands, guts and ovaries from partially engorged tick females were collected in RNAlater® (Ambion) and stored at −20 °C following the manufacturer’s protocol. Total RNA was extracted using the RNAqueous® Kit (Ambion) according to the manufacturer’s protocol and quantified by Qubit™ fluorometer. Two hundred nanograms of total RNA were utilized for cDNA synthesis using the Superscript® Vilo™ cDNA Synthesis Kit (Invitrogen) following the manufacturer’s protocol. Technical replicates were performed to evaluate enzymatic variations during the synthesis of cDNA in a given RNA sample. The *RmAQP2* cDNA sequence was used to design qPCR primers to amplify a fragment of 105 base pairs (Table 1). Quantitative real-time PCR were performed in a CFX96™ Real-Time PCR Detection System using the SsoFast™ EvaGreen® Supermix (Bio-Rad). Cycling conditions consisted of an enzyme activation step of 95 °C for 30 s followed by 40 cycles of denaturation at 95 °C for 5 s and annealing/extension of 60 °C for 5 s. Reactions were performed in duplicate in 20 μl using 200 nM of each primer and 2 μl of a 1/20 dilution of cDNA as template. The CFX Manager™ Software (Bio-Rad) was used to analyze the RT-qPCR data. Gene expression was normalized to the total amount of RNA used to generate the cDNA, as xpreviously described [13]. The *RmAQP2* transcription level was then calculated as a relative expression using the formula: Relative expression_(sample)_ = 2^[*Cq*(control) − *Cq*(sample)]^, where the control is either the highest or lowest C*q* value for the gene of interest, as previously described [13, 25]. Melt curve analyses showed the absence of primer dimers and nonspecific amplification. The absence of PCR product in no RNA control reactions and in no transcriptase reverse control reactions indicated the specificity of the *RmAQP2* RT-qPCR (data not shown).

### Monoclonal antibody production and immunobloting assays

Three synthetic peptides from the extracellular loops of RmAQP2 were manufactured by BioSynthesis, Inc. (Texas, USA) (Additional file 1: Figure S1). Peptide 1: LGSVGLAAAP (10-mer) (Amino acids 52 to 61). Peptide 2: ADALSQVDVNLAIVYGTNATAPVFSCFPAPGV (32-mer) (Amino acids 125 to 156). Peptide 3: MCGWGSAVFSFRSYNWFWV (19-mer) (Amino acids 229 to 247). Three 6-week-old BALB/c female mice were immunized subcutaneously with 50 mg of a pool of all three RmAQP2 peptides diluted in 0.1 ml of sterile PBS emulsified with an equal volume of TiterMax® Gold Adjuvant (Sigma-Aldrich). The primary immune response was boosted twice by subcutaneous immunization at 15-day intervals with the same concentration of peptide antigen plus adjuvant. Mice immune responses were monitored by ELISA using the RmAQP2 peptides as antigens. Three days prior to cell fusion, mice were boosted intravenously with the same concentration of antigen without adjuvant. Cell fusions and cloning by limiting dilution were performed by standard procedures [26]. Supernatants from the initial fusion and from clones obtained by limiting dilution were screened by ELISA. A total of 10 hybridoma clones were obtained and the hybridoma supernatants used to assess the expression of RmAQP2 in tick tissues and stages by immunoblot. For the immunoblot, antigens were prepared from unfed larvae, engorged nymphs, and salivary glands and guts of partially engorged females (at 5 days of feeding). Tick tissues and stages were homogenized in lysis buffer-Nonidet-P40 (NP-40) (150 mM sodium chloride 1.0 % NP-40 - 50 mM Tris, pH 8.0) and protease inhibitor (1 μg/ml). Total protein was quantified by Micro BCA Protein Assay (Thermo Scientific Pierce) and 3 μg of total protein were separated on 4–20 % Mini-PROTEAN® TGX™ Precast Gels (Bio-Rad) under reducing conditions. Proteins were transferred to a nitrocellulose membrane (Whatman, Dassel, Germany) for 1 h at 100 V. The membrane was blocked with 5 % skim milk in TBS (Tris-buffered saline: 25 mmol l^−1^ Tris–HCl, 150 mmol l^−1^ NaCl, pH7.6) for 1 h at room temperature, washed three times in TBS and incubated 1 h with the appropriate hybridoma supernatant as the primary antibody. The membrane was washed three times with TBS and incubated 30 min with anti-mouse HRP conjugated secondary antibody, then washed again three times with TBS and developed using chemiluminescent HRP antibody detection reagents.

### Synthesis of double stranded RNA

Synthesis of double stranded RNA (dsRNA) was performed as previously described [13]. Specific primers were designed based on the cDNA sequence of the *RmAQP2* gene described in the *R. microplus* Gene Index Project [17] (Table 1). Two fragments of approximately 400 bp were amplified, one located at the 5′ and another one located at the 3′ of the *RmAQP2* gene (Additional file 1: Figure S1). PCR products were cloned into pCR™II-TOPO® (Invitrogen), sequenced and used for *in vitro* transcription. The MEGAscript® Transcription Kit (Ambion) was used for the dsRNA synthesis following the manufacturer’s protocol. The two *RmAQP2* dsRNA molecules were checked by electrophoresis on agarose gel, quantified by spectrophotometry and kept at −20 °C until used for tick injection.

### Injection of ticks with double stranded RNA and assessment of gene silencing

Freshly molted unfed females were used for the dsRNA injection. Two independent experiments were run, each with a control group of 200 ticks injected with buffer alone and an experimental group with 200 female ticks each injected with either one or two dsRNA segments identical to *RmAQP2* (Additional file 1: Figure S1). For the first experiment, adult female ticks were injected with one segment of dsRNA from the 3′ end of the gene and fed on *B. bovis*-infected calf. For the second experiment, adult female ticks were injected with both segments of dsRNA, one from from the 5′ end and one from the 3′ end of the gene, then fed to repletion on an uninfected calf (*B. bovis*-free animal). Individual females were injected with 1 μl total dsRNA (approximately 1 × 10^11^ molecules dissolved in 0.1 mM EDTA buffer) or buffer control (0.1 mM EDTA buffer) through the coxal membrane at the base of the 4^th^ leg on the right ventral side, as previously described [13]. Ticks injected with the two different dsRNA segments received 0.5 × 10^11^ of each molecule for a total of 1 × 10^11^ molecules. Injections were accomplished using a 10 μl syringe with a 33 gauge needle (Hamilton, Bonaduz, Switzerland) and the microprocessor controlled UMP3 injection pump apparatus (World Precision Instruments, Berlin, Germany). After the injection, the dsRNA-injected females, plus an equal number of males, and the buffer-injected females, plus an equal number of males, were placed under separate stockinet sleeves glued to the calf to feed on either a *B. bovis*-infected calf (experiment 1) or an uninfected calf (experiment 2). For both experiments silencing level of *RmAQP2* was investigated using RT-qPCR as described above. Five days after the dsRNA injection, 20 partially engorged female ticks from each group were collected for dissection. Tissue collection, extraction of total RNA, cDNA Synthesis, and qPCR were performed as described above.

### Evaluation of tick fitness

After the dsRNA injection, individual stockinet sleeves were checked daily for the presence of engorged female ticks. Fully engorged females were collected, weighed and put in individual wells in 24-well plates at 26 °C for oviposition. At day 14 after the beginning of oviposition, egg masses laid by each individual female were weighed and put in individual vials to evaluate hatching. Hatching was evaluated at 30 days after the egg masses were weighed and hatching positive was defined as the presence of any larvae from eggs of an individual female. The larval progeny were maintained in individual vials at 26 °C for 45 days and the larval survival was determined as the presence of any live larvae among larval progeny from each individual female.

### Statistical analysis

Weights of engorged females and egg masses were compared by Student’s *t*-test (GraphPad Instat®, version 3.06, GraphPad Software, Inc., San Diego, CA, USA). The percentages of engorged females, oviposition, hatching and larvae survival were compared by Chi-squared (GraphPad Instat®). Gene expression in different tick tissues and stages was compared by one-way ANOVA and Tukey post-hoc test.

## Results

### *In silico* sequence analysis of the *R. microplus* AQP2

**Fig. 1:**
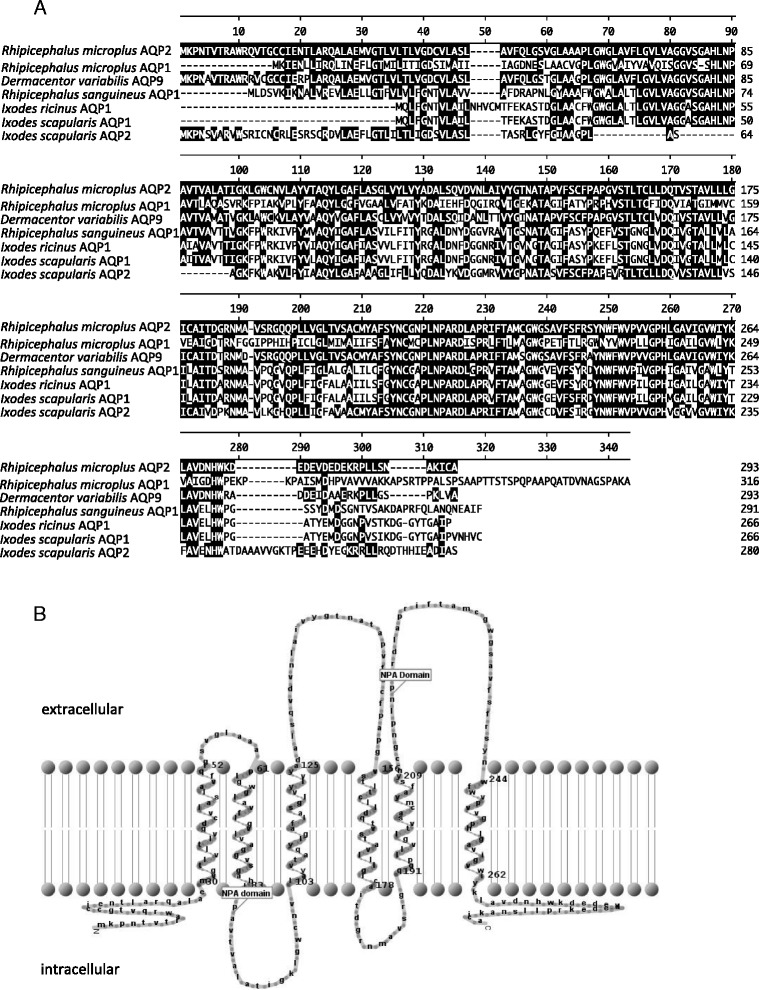
Multiple sequence alignments and topology prediction of RmAQP2. Panel **a** shows the amino acid alignment of RmAQP2 [GenBank ID: KP406519], *R. microplus* AQP1 [GenBank ID: KJ626366.1], Dermacentor *variabilis* AQP9 [GenBank ID: ABI53034.1], *Rhipicephalus sanguineus* AQP1 [GenBank ID: CAR66115.1], *Ixodes ricinus* AQP1 [GenBank ID: CAX48964.1], *Ixodes scapularis* AQP1 [GenBank ID: XP_002399532.1] and *Ixodes scapularis* AQP2 [GenBank ID: XP_002400655.1]. Black boxes in Panel A indicate identical amino acid residues when comparing RmAQP2 and the other sequences. Panel **b** shows the topology prediction of RmAQP2 indicating the presence of 6 transmembrane-spanning regions, 3 extracellular loops, 2 intracellular loops and 2 NPA AQP signature domains

Multiple alignment analysis revealed that the RmAQP2 amino acid sequence [GenBank ID: KP406519] presents 41.2, 86.0, 53.0, 53.5, 54.1, 65.5 percentage identity to *R. microplus* AQP1 [GenBank ID: KJ626366.1], *Dermacentor variabilis* AQP9 [GenBank ID: ABI53034.1], *R. sanguineus* AQP1 [GenBank ID: CAR66115.1], *Ixodes ricinus* AQP1 [GenBank ID: CAX48964.1], *I. scapularis* AQP1 [GenBank ID: XP_002399532.1] and *I. scapularis* AQP2 [GenBank ID: XP_002400655.1], respectively (Fig. 1). Considering domain organization, it is important to mention the presence of two AQP signature NPA motifs in the RmAQP2 sequence as well as in all the other AQP’s sequences mentioned above, except in the *I. scapularis* AQP2. The two AQP NPA motifs are present in RmAQP2 at the amino acid positions 84 to 86 and 216 to 218. Topology prediction indicates that RmAQP2 has six transmembrane-spanning regions and five loops; two loops are intracellular while the remaining three loops are extracellular. Therefore, the topology prediction of RmAQP2 is consistent with similar predictions previously done for other members of the AQP family (an *in silico* generated model of the predicted topology of the *R. microplus* AQP1 is shown in Fig. 1) [1, 2, 4]. Interestingly, this model also predicts the localization of the N- and C-terminal ends of RmAQP2 on the cytoplasmic side of the plasma membrane (Fig. 1), a characteristic feature of most known AQP proteins. Three-dimensional modeling [23] of the RmAQP2 protein suggests that it exists in the membrane as a tetramer.

### Differential pattern of transcription of the *RmAQP2* gene among distinct tissues and live stages of the tick

**Fig. 2:**
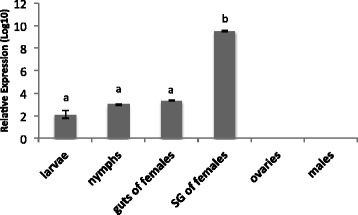
Expression of the *RmAQP2* gene in unfed larvae, engorged nymphs, unfed males, and salivary glands, gut and ovaries of partially engorged (day 5 of feeding) *R. microplus* females. Six biological replicates of unfed larvae (approximately 100 larvae per sample), engorged nymphs (approximately 10 nymphs per sample), unfed males (approximately 10 males per sample), and individual salivary glands, ovaries and guts of partially engorged females (at day 5 of feeding) were analyzed by reverse transcriptase quantitative real-time PCR. The transcription level of *RmAQP2* was calculated as relative quantity using the delta *C*_*t*_ comparative method normalized by the total amount of RNA used to generate the cDNA. Different letters (a, b) above the bars indicate significant statistical differences (one-way ANOVA, Tukey post hoc test, *P* < 0.001)

The transcription level of *RmAQP2* was investigated by RT-qPCR in different tissues and stages of *R. microplus* fed on uninfected calves (*B. bovis*–free calves) (Fig. 2). In a previous study we tested several reference gene candidates from different tissues and stages of *R. microplus,* but none of them was found adequate for normalization of gene expression [14]. Therefore, in this study the expression level of *RmAQP2* was normalized to the total amount of RNA used to generate the cDNA and the transcription level was calculated as a relative expression using the highest or lowest C*q* value for *RmAQP2* in a given sample as a control. Tick stages and tissues that were examined to evaluate gene expression included: unfed larvae (approximately 100 larvae per sample), engorged nymphs (10 nymphs per sample), unfed males (10 males per sample), and individual salivary glands, ovaries and guts of partially engorged females (at day 5 of feeding). We analyzed six biological replicates of each sample by RT-qPCR. Relative levels of expression of *RmAQP2* were different in larvae (2.5 [±0.66]), nymphs (3.0 [±0.23]), female guts (3.3 [±0.99]) and female salivary glands (9.5 [±1.73]) (Fig. 2). However, no transcripts were detected in ovaries of partially engorged females and unfed male ticks. The relative gene expression of *RmAQP2* in the female salivary glands samples was approximately 6 times higher (*P* < 0.001) than in larvae, nymphs and guts of females.

### Differential Expression of the RmAQP2 protein in different tick stages and tissues

**Fig. 3:**
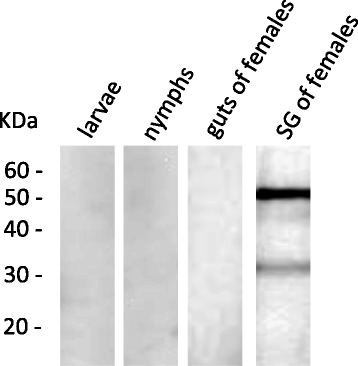
Immunoblot analysis demonstrating the pattern of expression of RmAQP2 in different tick life stages and tissues. Two bands at approximately 30 and 50 KDa were detected in salivary glands (SG) of partially engorged females. No protein band was detected in larvae, nymphs or guts of partially engorged females. Hybridoma supernatant 147-677-11 was used at a 1:3 dilution. Anti-mouse HRP conjugates were used at a 1:5,000 dilution. Assays were developed using chemiluminescent HRP detection reagents

Following the detection of *RmAQP2* mRNA in unfed larvae, engorged nymphs, and guts and salivary glands of partially engorged females, we investigated the presence of RmAQP2 protein in these tick stages and tissues (Fig. 3). Immunoblot assays were carried out using hybridoma supernatants. A hybridoma supernatant designated 147–677.13.11, used at a dilution 1:3, was selected for the immunoblot assay shown in Fig. 3. Anti-Bm86 polyclonal antibody was as a positive control used to demonstrate the presence of tick antigens in tick gut tissues for the immunoblot assays as previously described [27] (data not shown); however, we do not have a protein with associated antibody for protein expressed exclusively in the salivary gland as for aquaporin available for use as a salivary gland positive control. The aquaporin monoclonal antibody recognized proteins only in salivary glands of partially engorged females. In the blot shown in Fig. 3 shows that mAb 147–677.13.11 recognized a ≈ 30KDa and an apparently more reactive ≈ 50KDa protein. The monomeric form of the RmAQP2 protein has a molecular mass predicted from the sequence of ≈ 30 KDa, likely corresponding to the smaller ≈ 30KDa band recognized by the mAb in the blot. Based on structural predictions we suggest (see discussion) that the larger ≈ 50KDa band may represent an AQP dimer. Interestingly, the antibody tested in the immunoblots didn’t recognize any proteins in larvae, nymphs or guts of partially engorged females.

### Silencing of *RmAQP2* in salivary glands and its effect on protein expression

**Fig. 4:**
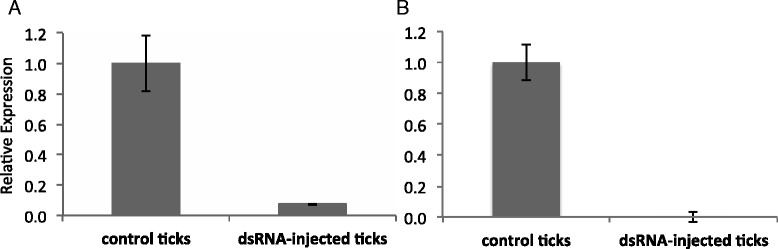
Transcript level of *RmAQP2* in salivary glands of partially engorged *R. microplus* females injected with dsRNA or buffer control. A total of 6 biological replicates were analyzed at day 5 after the double stranded RNA (dsRNA) injection. Panel **a** and **b** show the level of gene silencing of ticks injected with 1 fragment or 2 fragments of *RmAQP2* dsRNA, respectively. *RmAQP2* was silenced 92.8 % (±0.26 %) and 99.3 % (±3.70 %) in ticks injected with one dsRNA or two dsRNA fragments, respectively

**Fig. 5:**
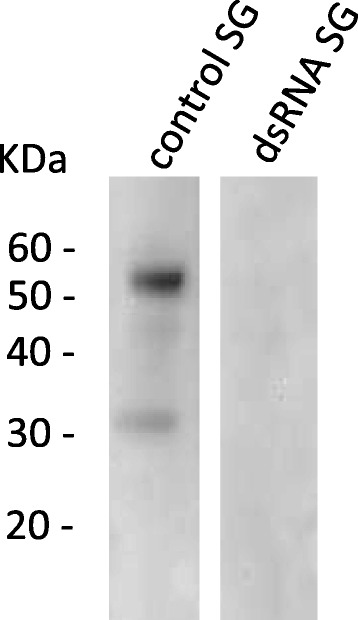
Immunoblot analysis demonstrating the effect of gene silencing on the expression of the RmAQP2 protein in salivary glands (SG) of partially fed female ticks. No protein was detected in salivary glands of dsRNA-injected ticks. Two bands at approximately 30 and 50 KDa were detected in salivary glands of the control non-silenced ticks. For the immunoblot assays, hybridoma supernatant 147-677-11 was at a 1:3 dilution. Anti-mouse HRP conjugate was used at a 1:5,000 dilution. Assays were developed using chemiluminescent HRP detection reagents

To assess *RmAQP2* silencing in salivary gland, female ticks were injected with dsRNA. For the first experiment, adult female ticks were injected with one segment of dsRNA from the 3′ end of the gene and fed on *B. bovis*-infected calf. For the second experiment, adult female ticks were injected with two segments of dsRNA, one from the 5′ end and one from the 3′ end of the gene, and then fed to repletion on an uninfected calf (*B. bovis*-free animal). Both dsRNA fragments were approximately 400 bp in length and their respective location on the *RmAQP2* gene is represented in Additional file 1: Figure S1. To assess silencing, salivary glands were examined from six individual ticks (representing 6 biological replicates) by RT-qPCR after 5 days of feeding on cattle. Similar levels of *RmAQP2* silencing were obtained when either one or two dsRNA fragments were used for silencing. *RmAQP2* was silenced 92.8 % (±0.26 %) and 99.3 % (±3.70 %) in ticks injected with either one dsRNA or the two non-overlapping dsRNA fragments, respectively (Fig. 4). Next, we investigate the effect of *RmAQP2* silencing on protein expression. Female ticks injected with two dsRNA fragments were fed on a calf for 5 days, dissected and individual salivary glands collected. Figure 5 demonstrates the absence of RmAQP2 protein in salivary glands of dsRNA-injected ticks. As expected, the presence of RmAQP2 protein was demonstrated in salivary glands of the control non-silenced ticks (Fig. 5). Collectively, the data demonstrated gene silencing and abrogation of protein expression as a consequence of injecting ticks with dsRNA identical to *RmAQP2*.

### Effect of *Rm*AQP2 silencing on tick fitness

After removal of the ticks for assessment of silencing and protein expression, the reminder of the ticks in the two independent experiments were allowed to feed to repletion to investigate the *in vivo* biological effect of *Rm*AQP2 silencing. For the first experiment, adult female ticks were injected with one segment of dsRNA (from the 3′ end of the gene) (Additional file 1: Figure S1) and fed on *B. bovis*-infected calf. For the second experiment, adult female ticks were injected with two segments of dsRNA (from both the 5′ and 3′ ends of the gene) (Additional file 1: Figure S1) and fed to repletion on an uninfected calf (*B. bovis*-free animal). In both experiments after the dsRNA-injected females were fed to repletion, the percentage of engorged females, weight of engorged females, oviposition rate, egg masses, percentage of hatching and percentage of larvae survival were evaluated (Tables 2 and 3). Considering the ticks that fed on a calf during acute *B. bovis* infection (experiment 1), identical numbers of females from both, the *RmAQP2* silenced group and control group, fed to repletion. The average weight of the *RmAQP2*-silenced engorged females was 361.8 mg and significantly higher (*P* < 0.05) than the control group (342.4 mg). Silencing of *RmAQP2* had no effect on the percentage of oviposition and egg masses between the two groups of ticks. In contrast, only 50 % of the egg masses hatched compared to 90.2 % hatching of the control group. Strikingly, none of the larvae survived in the *RmAQP2*-silenced group compared to 100 % larvae survival in the control group (Table 2). Considering the ticks that fed on the uninfected calf (experiment 2), identical numbers of females from both, the *RmAQP2* silenced group and control group, fed to repletion. The average weight of engorged females in the *RmAQP2* silenced group was 323.3 mg and significantly higher (*P* < 0.05) than the average weight of engorged females in the control group (305.6 mg). Oviposition rate was not affected by *RmAQP2* silencing; however, egg mass weight was significantly lower (*P* < 0.05) in the silenced group (134.7 mg) than in the control group (203.4 mg). Interestingly, the percentage of hatching was significantly reduced (*P* < 0.05) in the silenced group (81.2 %) compared to the control group (97.7 %); however, 100 % of the larvae survived in both the silenced and control groups (Table 3).

**Table 2:** Biological effect of *RmAQP2* silencing on tick fed on a calf during acute *B. bovis* infection (experiment 1)

	Percentage of engorged females	Weight (mg) of engorged females	Oviposition rate	Egg mass (mg)	Percentage of hatching	Percentage of larvae survival
Control females fed on a *B. bovis*-infected calf	46.1 % (83/180)	342.4 (±5.6)	98.7 % (82/83)	141.1 (±42.3)	90.2 %	100 % (74/74)
Silenced females fed on a *B. bovis*-infected calf	46.1 % (83/180)	361.8 (±7.0)^a^	96.3 % (80/83)	139.5 (±50.5)	50.0 %^b^	0

^a^ *t* test (*P* < 0.05)

^b^Chi-squared test (*P* < 0.05)

**Table 3:** Biological effect of *RmAQP2* silencing on tick fed on an uninfected calf (experiment 2)

	Percentage of engorged females	Weight (mg) of engorged females	Oviposition rate	Egg mass (mg)	Percentage of hatching	Percentage of larvae survival
Control females fed on an uninfected calf	37.7 % (68/180)	305.6 (±6.1)	98.5 % (67/68)	203.4 (±39.0)	97.7 %	100 %
Silenced females fed on an uninfected calf	51.1 % (92/180)	323.3^a^ (±5.7)	94.5 % (87/92)	134.7^a^ (±63.6)	81.2 %^b^	100 %

^a^ *t* test (*P* < 0.05)

^b^Chi-squared test (*P* < 0.05)

## Discussion

In the present study we investigated the pattern of expression of a newly identified aquaporin 2 of *R. microplus* ticks. *RmAQP2* transcript was demonstrated in unfed larvae, engorged nymphs, and salivary glands and guts of partially engorged female ticks. Interestingly, however, among the ticks tissues and stages investigated, RmAQP2 protein was found only differentially expressed in the salivary glands of partially engorged females. *In vivo* functional analyses showed that silencing of *RmAQP2* decreased tick fitness and this effect was more pronounced in ticks fed on a calf during acute *B. bovis* infection. Noteworthy, none of the larval progeny from the *RmAQP2*-silenced females that were fed on a *B. bovis*-infected calf survived. Considering that *R. microplus* larvae transmit *B. bovis*, these data may have implications for the control of tick-borne parasites [6].

Since *R. microplus* is a one-host tick that infests the host as larvae and remains until the adult females have fed to repletion, we did not think it was meaningful to assess all tick life stages of the tick for aquaporin expression. The adult female takes the majority of the blood. It is that stage that is primarily responsible for acquisition of *B. bovis* and it is also that stage that causes the most direct damage to the host by feeding. *B. bovis* is passed from the adult female transovarially to the larval offspring and it is the larval stage that transmits *B. bovis*. Consequently, the most biologically relevant stages of the tick for acquisition and transmission of *B. bovis* are the adult female and the larvae, respectively. Adult females are the most biologically relevant stages with regard to damage cause by tick feeding. Therefore, our investigations focused primarily on these two stages for expression of the aquaporin gene.

Members of the AQP family have recently been identified in several arthropods including tick species, such as *D. variabilis*, *R. sanguineus*, *I. ricinus*, *I. scapularis* and *I. scapularis* [28, 29]. Here we described a newly identified AQP2 of *R. microplus* ticks. The presence of two NPA domains and the level of amino acid identity between RmAQP2 and other AQPs prompt us to argue about a potential evolutionary conserved function for these proteins. The remarkable similarity of topology among AQP members in different species, including RmAQP2 described in this study, also supports the prediction of a putative conserved function [1, 2, 4]. A cysteine located five residues upstream of the second NPA motif suggests that RmAQP2 is likely to be sensitive to mercury as demonstrated for other AQP members [1]. This may set the rationale for the design of novel acaricides targeting AQP to control *R. microplus* infestation and *R. microplus*-borne parasites. It has been proposed that AQPs play a critical osmoregulatory function in blood-feeding arthropods during saliva production, intake and excretion of water during blood digestion, reproduction and egg development, and off-host environment stress tolerance [3]. Salivary glands are of vital importance for tick biology, acting as osmoregulatory organs for both off-host and on-host stages of their life cycle [29]. We postulate that AQPs expressed uniquely in the tick salivary glands may play an important role in the osmoregulatory stress during blood feeding and digestion. *RmAQP2*-silenced female ticks had a significant increase in body weight at the time of repletion compared to control non-silenced ticks, suggesting a level of involvement of *RmAQP2* in osmoregulatory mechanisms. Additionally, the increased body weight implies that ticks accumulate more liquids in the absence of RmAQP2. Despite being heavier, the *Rm*AQP2-silenced group produced egg masses of reduced weight and reduced hatching success when compared with the control group, suggesting a negative correlation between intake of blood and generation of viable larval offspring.

Considering that RmAQP2 protein was only found in salivary glands, it is reasonable to assume that this AQP plays an essential role in osmoregulation during blood feeding and digestion. The data showed that knockdown of *Rm*AQP2 interfered with the osmoregulatoty system of *R. microplus* during feeding as demonstrated by the increased body weight in the gene-silenced engorged females. Effect on tick fitness was even more pronounced in ticks fed on a *B. bovis*-infected calf perhaps due to the deleterious presence of the apicomplexa parasite infection [6]. Additionally, it is reasonable to speculate about a potential cumulative effect of *RmAQP2* silencing throughout the tick life cycle considering that none of the larval progeny survived from the silenced females that fed on the calf during acute *B. bovis* infection. Here we also show that dsRNA injection abolished the expression of RmAQP2 protein completely in salivary gland of partially engorged females at 5 days of feeding. This result suggests a possible rapid turnover of RmAQP2 in salivary glands. Rapid turnover of AQPs has been previously demonstrated both in animal and plant tissues and shown to be more pronounced during infections or when organisms are under stress [30–32]. The extreme osmoregulatory changes in salivary glands of ticks during feeding could induce a high level of physiological turnover of AQPs, as demonstrated by the present data for RmAQP2. It was recently demonstrated that *AQP1* mRNA of *I. ricinus* ticks is expressed only in female tissues involved in water flux, such as guts, rectal sac and especially abundant in salivary glands [29]. Here we demonstrated a similar pattern of expression for *RmAQP2* transcripts. However, RmAQP2 protein was found in salivary glands. Therefore, it is plausible to assume the presence of a mechanism to suppress translation of *RmAQP2* in female guts, larvae and nymphs. Silencing of *I. ricinus AQP1* affected feeding performance, but the ticks remained feeding on the host with subsequent potential for pathogen transmission. Therefore, the authors concluded that *I. ricinus AQP1* is not a suitable target to control tick infestation or block transmission of tick-borne diseases [29]. Our data showed that *RmAQP2*-silenced female ticks ingested more blood and consequently showed increased engorgement body weight. Hatching and larvae survival were also affected by gene silencing and most importantly, none of the larval progeny from the *RmAQP2*-silenced females fed on a *B. bovis*-infected calf survived. Differences in site and level of expression, and permeability may explain the discrepancies between the present study and the previous published data regarding *I. ricinus AQP1* [29]. Another recent study evaluated AQP1 of *R. microplus* as an anti-tick vaccine [16]. The authors demonstrated that vaccination of cattle with recombinant *R. microplus* AQP1 effectively reduced the number of adult female ticks that fed to repletion.

Based on the amino acid sequence, RmAQP2 has a predicted molecular weight of ≈ 30 KDa. However, preliminary *in silico* analysis using NetOGlyc [33] and NetPhos [34] revealed the presence of several putative glycosylation and phosphorylation sites on RmAQP2 (data not shown). Post-translational modifications of AQP have been previously described [35–37] and it may be one explanation for the detection of larger protein products in the immunoblot assays. Structural predictions suggest that this protein may exist in the membrane as a tetramer and the multiple banding pattern in the blot could represent varying stages of the breakdown of this tetrameric structure; cleavage of the intracellular tails of the RmAQP2 would result in a molecular mass of ≈ 25 KDa, suggesting that the larger 50 KDa band could be a dimer of two molecules. In additional blots (not shown) we also see another band above 100 KDa, which could represent the tetramer. Another possibility to explain the difference between expected and observed molecular weight is that the *RmAQP2* cDNA sequence obtained from the *R. microplus* gene index project may be incomplete at the 5′ end. This sequence was derived from an EST project and although it has two stop codons in the 3′ end of the sequence, a stop codon in-frame with the presumed start ATG codon is not present. Nevertheless, this possibility is unlikely considering that the topology analyses revealed a typical predicted conformation for the AQP family [3, 4, 28]. Regardless of these considerations, the key finding is that all of the bands recognized by the monoclonal antibody raised to the RmAQP2 peptides are absent in the silenced ticks, suggesting that silencing has completely abrogated protein expression. It was beyond the scope of this study to determine the type of cell(s) in the tick salivary glands expressing the RmAQP2 protein; however, the immunoblot data clearly demonstrate that salivary glands are the unique site of expression of this AQP in *R. microplus*.

RNA interference has been widely used to investigate gene function in arthropods. However, off-target effects have been described in some species [38] and cannot be entirely ruled out as the cause of the results observed in this experiment. The *R. microplus* genome sequence is not available and the alignment analyses are restricted to sequences listed in databases. Blast analysis of the dsRNA sequences used in this study did not reveal significant homology to any known tick sequence other than *RmAQP2*. Additionally, gene silencing was validated by RT-qPCR and *RmAQP2* was silenced at similar levels when we used either one or two different segments of dsRNA identical to the target gene. Taken together, these aspects support the data and represent the best possible strategy to make solid scientific observations regarding the biological effect of *RmAQP2* silencing in *R. microplus*.

## Conclusion

Here we demonstrate the pattern of expression of a newly identified aquaporin 2 gene of *R. microplus* ticks. *RmAQP2* transcripts were present in unfed larvae, engorged nymphs, and salivary glands and guts of partially engorged female ticks. However, among the distinct adult tissues and life stages tested, RmAQP2 protein was only found in salivary glands of partially engorged females. *In vivo* investigation demonstrated that silencing of *RmAQP2* decreased tick fitness. The effect of *RmAQP2* silencing on tick fitness was even more pronounced in females fed on a *B. bovis*-infected calf and none of their larval progeny survived. Therefore, we conclude that RmAQP2 is involved in osmoregulation during feeding and is a potential target for the development of novel strategies for the control of *R. microplus* and *R. microplus*-borne diseases.

## Additional file

Additional file 1: Figure S1. Sequence of *RmAQP2* cDNA [GenBank ID: KP406519]. Blue and green lines indicate the 5′ double stranded RNA (dsRNA) (Blue) and 3′ dsRNA segments (green), respectively. Red lines indicate the regions utilized to design synthetic peptides for monoclonal antibody production. (PDF 66 kb)
