# Supplementary figures and images for: Targeted silencing of the Aquaporin 2 gene of Rhipicephalus (Boophilus) microplus reduces tick fitness
Source: Parasit Vectors. 2015 Dec 2;8:618. doi: 10.1186/s13071-015-1226-2 (PMC4667534; doi:10.1186/s13071-015-1226-2)

Figure 1S

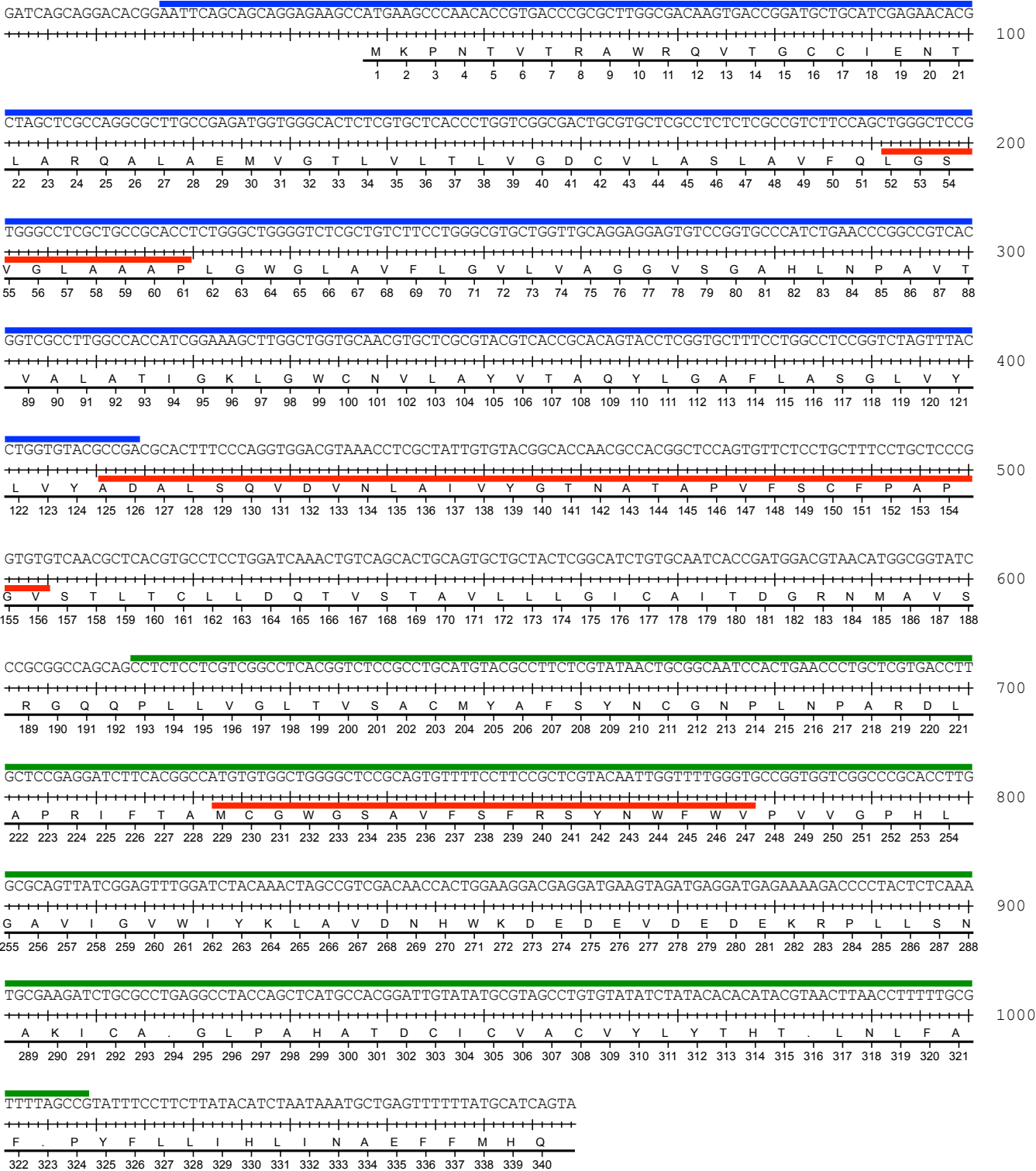

Supplement: Additional file 1: Figure S1. — Sequence of RmAQP2 cDNA [GenBank ID: KP406519]. Blue and green lines indicate the 5′ double stranded RNA (dsRNA) (Blue) and 3′ dsRNA segments (green), respectively. Red lines indicate the regions utilized to design synthetic peptides for monoclonal antibody production. (PDF 66 kb) [file 13071_2015_1226_MOESM1_ESM.pdf]
